# Highly conserved *Plasmodium vivax* genomes in Duffy-negative individuals from Sudan

**DOI:** 10.21203/rs.3.rs-7123704/v1

**Published:** 2025-08-07

**Authors:** Regan E. Schroeder, Safaa Ahmed, Anthony Ford, Mohammed Elfaki, Samuel Omer Hamad, Tarig Mohamed Elfaki Ahmed, Sumaia Mohamed, Emilia Manko, Taane G. Clark, Susana Campino, Muzamil Mahdi Abdel Hamid, Eugenia Lo

**Affiliations:** 1 Department of Microbiology and Immunology, College of Medicine, Drexel University, Philadelphia, USA; 2 Institute of Endemic Diseases, University of Khartoum, Khartoum, Sudan; 3 Department of Zoology, Faculty of Science, University of Khartoum, Khartoum, Sudan; 4 Department of Bioinformatics and Genomics, University of North Carolina, Charlotte, NC, USA; 5 Department of Basic Medical Sciences, Faculty of Medicine, Jazan University, Jazan, Saudi Arabia; 6 National Malaria Control Program, Federal Ministry of Health, Khartoum, Sudan; 7 Department of Infection Biology, London School of Hygiene and Tropical Medicine, Keppel Street, London, UK

**Keywords:** *P. vivax*, Duffy Blood Group, Sudan, Whole Genome Sequencing, Single Nucleotide Polymorphism, Epidemiology

## Abstract

Duffy-negatives were previously thought to be immune to *Plasmodium vivax* infections due to Duffy binding protein’s (PvDBP1) inability to invade erythrocytes lacking Duffy antigen receptor for chemokines (DARC) expression. Nevertheless, reports of *P. vivax* cases are growing throughout Africa and among Duffy-negative people. Although there are alternative invasion mechanisms by *P. vivax*, the exact mechanisms in Duffy-negative individuals are unclear. Sudan, with a mixed Duffy-negative and Duffy-positive population, is ideal to study differences between these infections on epidemiological and genetic scales. The goal of this study was to compare Duffy-positive and Duffy-negative infections in Sudanese individuals on epidemiological and genomic scales. We collected epidemiological data and sequenced parasite genomes and found that Duffy-positive individuals had significantly higher parasitemia than Duffy-negatives (p=0.0000132). Furthermore, Duffy-positive infected *P. vivax* genomes were much more diverse than Duffy-negatives, across all 14 chromosomes and 44 specific erythrocyte binding gene candidates. Genes of the merozoite surface protein family account for much of the genetic diversity found. Many erythrocyte binding gene candidates are under selection pressure, both positive and negative. Finally, in genes currently implicated in binding, we have found amino acids that have undergone mutations to a structurally different residue, potentially affecting binding and antigenic conformation.

## Introduction

*Plasmodium vivax*, the second most prevalent malaria parasite in humans, has long been considered as a less severe counterpart to the more deadly *P. falciparum*^1,2^. However, emerging evidence demonstrates that *P. vivax* can cause severe malaria syndromes and substantial morbidity, challenging its historical perception as a minor disease^3,4^. While *P. vivax* is most prevalent in Asia and the Western Pacific, as well as in Central and South America^5^, it also has been widely reported in Africa, particularly in East Africa, like Sudan, Somalia, and Ethiopia, and increasingly common in Central and West Africa^6,7^. Sudan, a high-burden malaria region, has been previously reported to have ~17% of *P. vivax* infections in Duffy-negative individuals, though most of the infections occurred in Duffy-positive individuals. Such patterns suggest alternative erythrocyte invasion mechanisms, perhaps independent of Duffy status.

The invasion of host reticulocytes by *P. vivax* relies on interactions between its Duffy binding protein (PvDBP1) and the Duffy antigen receptor for chemokines (DARC) expressed on the red blood cell surface^8–10^. Due to a T>C mutation at the −67 position in the DARC promoter region, the receptor has little to no expression in the mature RBCs of Duffy-negative individuals and thus was thought to be immune to *P. vivax*^11,12^. However, recent studies challenge this conventional dogma, reporting several *P. vivax* cases in Duffy-negative individuals, raising significant public health concerns in Africa where Duffy-negative individuals are predominant^13–15^.

The mechanism by which *P. vivax* invades Duffy-negative red blood cells remains unclear^14^. *P. knowlesi*, often used as a surrogate for *P. vivax* in *in vitro* studies due to the latter’s inability to be cultured^16^, is unable to invade Duffy-negative red blood cells, thereby challenging the role of the PkDBP-DARC interaction in mediating invasion of Duffy-negative erythrocytes^17,18^. Furthermore, recent findings showed that *P. vivax* may exploit transient DARC expression on erythroblasts in Duffy-negative individuals, enabling erythrocyte invasion^19^. However, a few alternative, DARC-independent, pathways including PvTRAg38 binding to Band 3^20^, PvRBP2b to transferrin receptor 1 (TfR1)/CD71^21,22^, PvRBP2a to CD98^23^, PvEBP/DBP2 to Complement Receptor 1 (CR1)^24^, and PvAMA1 to PvRON2^25,26^ have also been shown to be involved in erythrocyte invasion. It is possible that genetic polymorphisms in these parasite ligand proteins, especially those unique in *P. vivax* from Duffy negatives, may alter erythrocyte binding and invasion efficiency^27^.

*P. vivax* is geographically structured and genetically diverse^28,29^. Distinct differences have been shown between isolates of different continents and further subgroupings within continents^28^. Although African *P. vivax* parasites are most similar to the South Asian isolates, there is distinct separation between the two regions^1,28^. Furthermore, *P. vivax* parasites are very genetically diverse, much more so than *P. falciparum*, making them less susceptible to population bottlenecks in low transmission areas^29,30^. In East Africa, *P. vivax* isolates were shown to be clustered by country, a pattern also seen in South Asia^31^. Parasite gene flow is limited not only by geographical distance, but also by landscape factors such as elevation and land cover at a fine scale^32^. Our previous study based on microsatellites has shown that *P. vivax* in Duffy-negatives and Duffy-positives were genetically similar, and that gene flow occurred frequently between the two populations^32^, suggesting that Duffy-negatives are not dead-end hosts but instead allow for transmission^32,33^. This study investigated the epidemiological trends of *P. vivax* infections in Sudan and compared single nucleotide polymorphisms (SNPs) of 14 chromosomes as well as 44 erythrocyte-binding gene candidates based on whole genome sequencing (WGS) between Duffy-negative and Duffy-positive infections. Findings offer insights into genetic mechanisms underlying the ability of *P. vivax* to infect Duffy-negative individuals in Africa.

## Results

### Characteristics of *P. vivax* Infections

**Figure 1:**
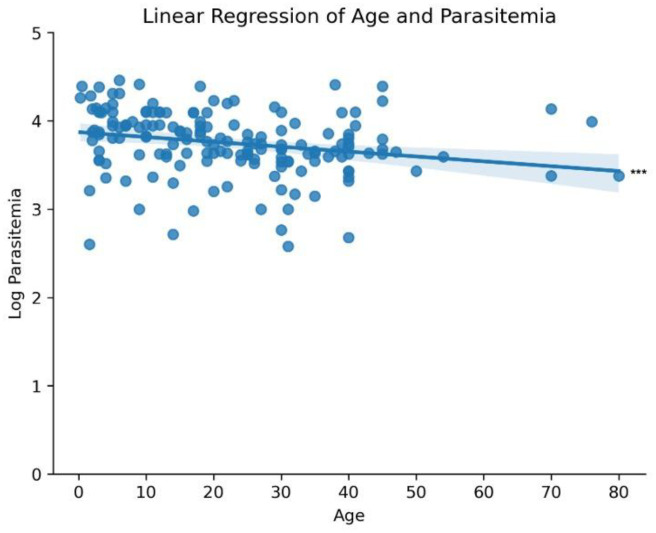
Parasitemia Incidence and Distribution by Age Group Heatmap depicting the incidence of parasitemia across different age groups. Linear regression showing a statistically significant correlation between age on the x-axis and log-transformed parasitemia on the y-axis. Shaded area represents error of the regression. *** p<0.001 (p=0.0000219) using Spearman correlation.

**Figure 2:**
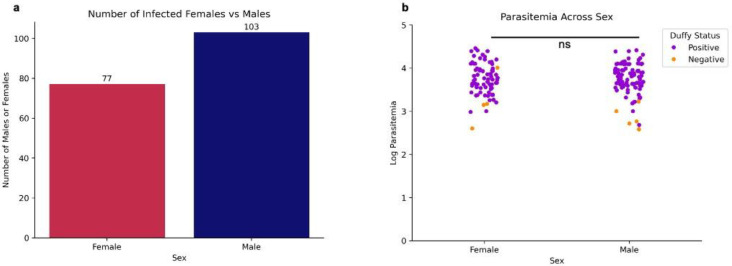
*P. vivax* incidence and parasitemia levels across sex Bar chart depicting the number of mono-infected female vs. male patients. There are significantly more cases of males being infected than females (A). Strip plot depicting parasitemia levels across female and male patients, of which there is no significant difference between the two (B). ns no significance (p=0.796) using Mann-Whitney U test.

**Figure 3:**
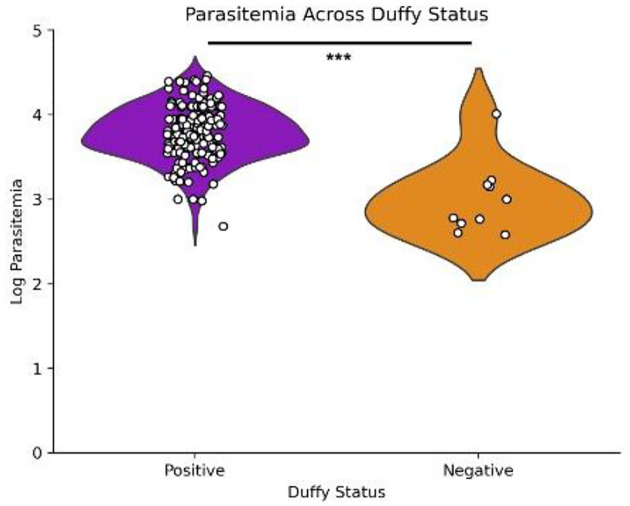
Parasitemia Levels by Duffy Status Violin plot depicting the distribution of log-transformed parasitemia levels in Duffy-positive (purple) and Duffy-negative (orange) individuals. Each point represents an individual sample, with higher density areas indicating a greater number of observations. Duffy-positive individuals exhibit a broader range of parasitemia compared to Duffy-negative individuals, suggesting a potential difference in susceptibility or parasitemia burden between the two groups. Duffy-positive individuals exhibit significantly higher P. vivax parasitemia as compared to Duffy-negative individuals when experiencing mono-infection. *** p<0.001 (p=0.0000132) using Mann-Whitney U test

Of the 238 blood samples obtained from suspected malaria patients, 187 were confirmed as *P. vivax* mono-infection by PCR and/or microscopy. Patient age ranged from 3 months to 80 years, and higher parasitemia levels were associated with younger age groups, with younger individuals demonstrating the highest incidence rates. A strong negative correlation was observed between age and parasitemia (Spearman correlation=−0.325, *p*=0.0000219; Figure 1). Despite differences in sample size, there was no significant difference in *P. vivax* parasitemia among study sites (Figure S2B) and by years (Figure S4). We observed more positive infections in males than females (Figure 2A) and parasitemia did not differ between genders (Figure 2B; *p*=0.796). Most *P. vivax* samples were detected in Duffy-positives (*n*=173), much higher than in Duffy-negative (*n*=10). Also, Duffy-positive samples exhibited significantly higher parasitemia than the Duffy-negative ones (*p*=0.0000132 by Mann-Whitney U test), despite a few outliers in the Duffy-positive group that showed considerably lower parasitemia (Figure 3).

### Genetic Polymorphisms of *P. vivax* between Duffy Groups

**Figure 4:**
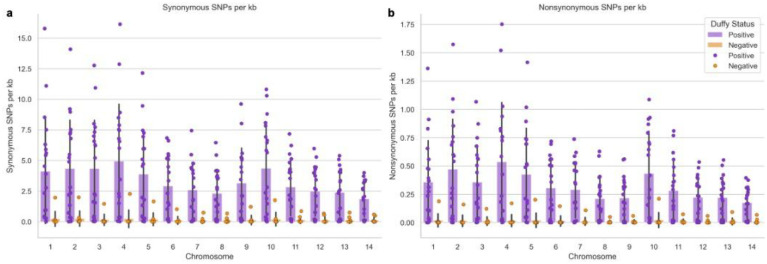
Synonymous and Nonsynonymous SNPs across 14 Chromosomes in Duffy Positive and Negative Individuals Bar charts depicting synonymous (A) and nonsynonymous (B) SNPs in Duffy-positive (purple) and Duffy-negative (orange) individuals. Each x-tick represents one of the 14 nuclear P. vivax chromosomes and the y-axis represents the density of SNPs in each chromosome depicted as SNPs per kb of DNA. There are significantly more of both synonymous and nonsynonymous SNPs in the Duffy-positive samples compared to the Duffy-negative samples across all chromosomes (Supplemental Table 2). Furthermore, there are significantly more synonymous than nonsynonymous SNPs in both Duffy-positive and Duffy-negative samples (Supplemental Table 2).

Whole genome sequence (WGS) data from a subset of 30 samples (Supplemental Table 1) including 10 from Duffy-negative and 20 from Duffy-positive indicated a significantly higher (Supplemental Table 2) number of synonymous and nonsynonymous single nucleotide polymorphisms (SNPs) in Duffy-positive compared to Duffy-negative isolates (Figure 4). This difference is exacerbated by the lower coverage among the Duffy-negative samples, where the average coverage across the 14 chromosomes ranges from 0.335- to 49.872-fold with a median of 1.565-fold (Supplemental Table 3). This is in stark contrast to the Duffy-positive samples where average coverage ranges from 0.592- to 96.290-fold with a median of 71.925-fold (Supplemental Table 3). Despite this, all SNPs identified passed stringent quality filtering. Both Duffy groups exhibited significantly more synonymous than nonsynonymous SNPs (Supplemental Table 2). Chromosomes 3, 4, and 10 had the highest average SNP density (number per kb) in Duffy-positive *P. vivax* for synonymous (4.335, 4.951, and 4.357 SNPs/kb, respectively) mutations, and 2, 4, and 10 for nonsynonymous (0.472, 0.536, and 0.433 SNPs/kb, respectively) mutations (Figure 4, Supplemental Table 4). In Duffy-negative *P. vivax*, the highest average SNP density was observed in chromosomes 1, 2, and 4 for synonymous SNPs (Figure 4A, Supplemental Table 4; 0.229, 0.258, and 0.238 SNPs/kb, respectively) and 1, 5, and 10 for nonsynonymous SNPs (Figure 4B, Supplemental Table 4; 0.0204, 0.0212, and 0.0228 SNPs/kb, respectively). Across the 14 chromosomes, PIR proteins were most prevalent among the most nonsynonymous SNPs in any given gene in each chromosome for both Duffy-positive and Duffy-negative samples (Supplemental Table 5). Notable exceptions include reticulocyte binding protein 2c (PvRBP2c), which is highly polymorphic in both Duffy-positive and Duffy-negative samples on chromosome 5; merozoite surface proteins 3.5 and 3.8 (PvMSP3.5 and PvMSP3.8), showing polymorphism in Duffy-positive and Duffy-negative samples respectively; and the DNA2/NAM7 helicase on chromosome 8, which is polymorphic in Duffy-negative samples (Supplemental Table 5). Other merozoite surface proteins account for 10 more of the most prevalent genes among the Duffy-positive genomes when looking at the 10 most nonsynonymous SNPs per chromosome, and 3 for the Duffy-negatives (Supplemental Table 5).

**Figure 5:**
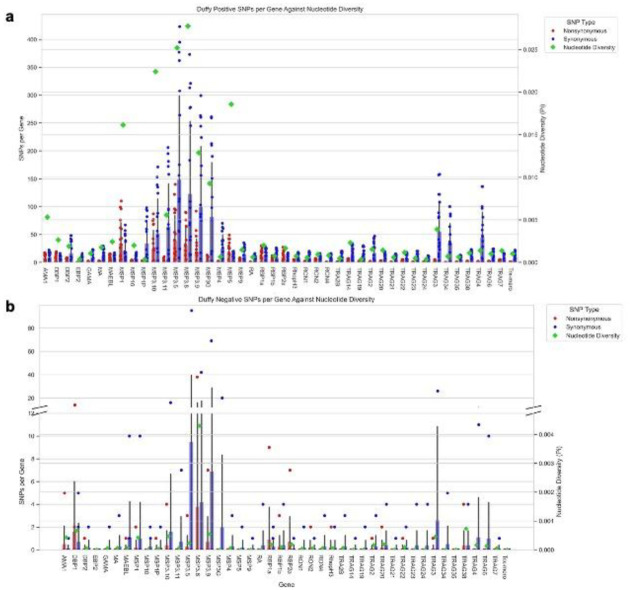
SNPs and Nucleotide Diversity in 44 Potential Erythrocyte Binding Genes Across Duffy-positive and Duffy-negative individuals Bar charts depicting the number of synonymous (blue) and nonsynonymous (red) SNPs in both Duffy-positive (A) and Duffy-negative (B) samples alongside nucleotide diversity (lime) of each gene. Across all 44 genes, nucleotide diversity is significantly higher in the Duffy-positive samples than the Duffy-negative samples (p<0.001 using Mann-Whitney U test). 27% of genes have no significant difference between the number of nonsynonymous mutations between Duffy status. 93% of Duffy-positive genes had significant differences in the amount of SNPs based on SNP type. Due to low coverage, no significance was detected across SNP types for Duffy-negative samples (Supplemental Table 6).

The analysis of the 44 potential erythrocyte binding gene (EBP) candidates revealed high genetic diversity in the *PvMSP* gene family, particularly *PvMSP*3.5 and *PvMSP3.8* for both Duffy-positive (p=0.025 and 0.028; Figure 5A) and Duffy-negative (p=0.0002 and 0.004; Figure 5B) samples. Additionally, *PvTRAg* genes such as *PvTRAg*3 exhibited relatively high diversity (p=0.004 in Duffy-positives, p=0.0004 in Duffy-negatives). Consistent with the genome-level variations, Duffy-negative samples were more conserved with fewer SNPs and lower nucleotide diversity in the 44 EBP genes than Duffy-positive ones. Similarly to samples on the chromosome level, there were significantly more SNPs in the Duffy-positive genes than Duffy-negative ones for both synonymous and nonsynonymous mutations for most genes (Supplemental Table 6). Exceptions to this include *PvEBP2, PvRA, PvRON4, PvTRA2B, PvTRAG2, PvTRAG21, PvTRAG23, PvTRAG24, PvTRAG34, PvTRAG35, PvTRAG38, and PvTRAG6*, where there was no significant difference between nonsynonymous SNPs across Duffy-status. Furthermore, for all genes except *PvDBP1, PvMAEBL*, and *PvRBP1a*, there were significantly more synonymous than nonsynonymous mutations in the Duffy-positive samples. No conclusions can be made for differences of mutation type in the Duffy-negative samples as low coverage prevented accurate statistical analysis (Supplemental Table 6).

*PvMSP3.5, PvMSP1*, and *PvMSP3.8* account for the most average nonsynonymous SNPs across all Duffy-positive (Supplemental Table 7; 39.3, 34, and 30.7 average SNPs per kb, respectively) samples. In the Duffy-negative, the most average nonsynonymous SNPs belong to *PvMSP3.8, PvDBP1*, and *PvRBP1a* (Supplemental Table 7; 3.8, 1.6, and 0.9 average SNPs per kb, respectively), although these data may be skewed due to the low coverage of the Duffy-negative samples. This low coverage also lead to the Duffy-negative samples having a median dN/dS value of 0 for all 44 EBPs (Table 1). Median values were used instead of means to diminish the effects of outlying values. The Duffy-positive samples, however, has multiple genes showing selection pressure, as there are multiple genes where median dN/dS>1 (*PvAMA1, PvDBP1, PvMSP3.5, PvMSP3.10, PvMSP5, PvRBP1a, PvRBP1b*, and *PvRBP2a*), indicating positive selection pressure, and where dN/dS<1 (*PvMAEBL, PvMSP1, PvMSP3.8, PvMSP3.9, PvMSP3G, PvMSP9, PvMSP10, PvTRAG3, PvTRAG14*, and *PvTRAG20*; Table 1), potentially indicating negative selection pressure. The rest of the 44 EBPs have median dN/dS values of 0, resulting from a 0 in either dN or dS—from a lack of either nonsynonymous or synonymous mutations (Table 1).

**Figure 6:**
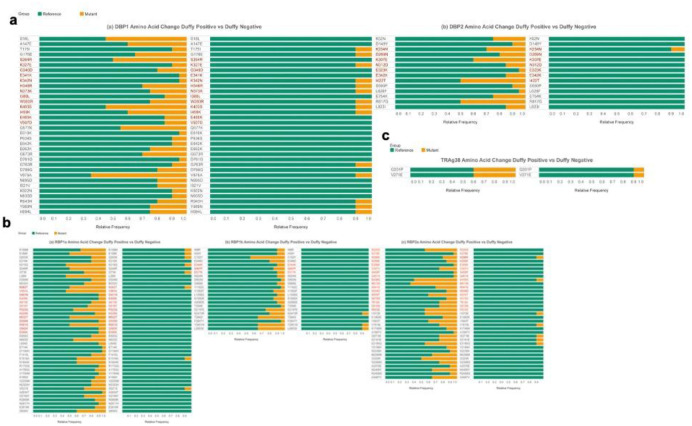
Amino Acid Mutation Prevalence in 7 EBPs Across Duffy-Status Horizontal stacked bar plots showing the reference codon (green) prevalence against mutant codon (yellow) prevalence for each nonsynonymous mutation found in the gene for both Duffy-positive and Duffy-negative samples. Significance of each codon mutation is presented in Supplemental File 1. The x-axis represents the frequency of each nonsynonymous mutation or reference amino acid across all 20 (Duffy-positive) or 10 (Duffy-negative) samples. Y-axis represents individual codon mutations found in the genomes. Represented genes include duffy binding proteins (A), reticulocyte binding proteins (B), and tryptophan rich antigen 38 (C). Many amino acid mutations were

At the codon level, a much higher prevalence of amino acid mutations was detected in the Duffy-positive than the Duffy-negative samples (Figure 6). Among Duffy-positive samples, 13 mutations were found in the binding region of *PvDBP1*, with more than 50% of samples containing the amino acid change Lys403Ser. In Duffy-negative samples, only seven mutations were found in the binding region, with no more than 10% of samples containing the amino acid change at these seven positions (Figure 6A(a)). Fewer mutations were observed in the binding region of *PvDBP2/EBP*, with seven observed in Duffy-positive samples and only one observed in Duffy-negative samples.

The mutation rate ranged from 10% to 50% in the Duffy-positive samples, where the most prevalent mutation was Ile422Thr, with in contrast, only 10% of samples containing the Lys234Asn mutation in the Duffy-negative samples (Figure 6A(b)). Similar mutation rates are observed in *PvRBP1a*, with 13 mutations found in Duffy-positive individuals (10% to 50% of samples containing these mutations), most prevalently Val391Ala, and only one mutation (Asn362Thr) found in Duffy-negative individuals (10% of samples containing the mutation; Figure 6B(a)). *PvRBP1b* contained three binding region mutations in the Duffy-positive samples all at low (<20%) prevalence, and none were found in the Duffy-negative samples (Figure 6B(b)). *PvRBP2a* contained 17 mutations in the binding region in Duffy-positive samples, with 10% to higher than 60% of samples containing mutations, where the mutation present in the most samples is Met512Lys. Two mutations in the binding region of *PvRBP2a* were found in the Duffy-negative samples, each present in 10% of the samples (Figure 6B(c)). *PvMSP1* had the most mutations, with 192 total mutations and 108 mutations (10% to less than 50% of samples contained the amino acid mutations) within the binding region, all within Duffy-positive samples. Only one mutation was found in the Duffy-negative samples with only 10% of samples containing the mutation (Supplemental Figure 5). PvTRAg38 had the fewest mutations with only two regions having an amino acid substitution, none of which were found in the binding region (Figure 6C). The mutation was present in 40% of Duffy-positive samples and 10% of Duffy-negative samples.

## Discussion

This study investigated *P. vivax* epidemiology across different transmission settings of Sudan and genetic variations of *P. vivax* isolates from Duffy-positive and Duffy-negative Sudanese people. Like other East African countries, Duffy-positives and Duffy-negatives coexist in Sudan, and *P. vivax* infections have been previously reported in both populations^13,34^. No significant difference was detected in parasitemia by transmission setting. Despite broader regional trends showing increased malaria cases and deaths during the COVID-19 pandemic^35^, parasitemia across samples from 2018, 2019, and 2021 did not significantly differ. Age was negatively correlated with parasitemia and we detected more males than females with *P. vivax* infections. In high endemic areas, children are disproportionately affected by *Plasmodium* infection^36^. Children 6 months to five years of age are most at risk for malaria because this is the time period where they lost maternal immunity without their own immune memory developed yet^37^. Apart from age, febrile symptoms and other host factors, such as Duffy blood group, could also affect parasitemia^38–40^. Our analyses indicated that Duffy-positive *P. vivax* infections had significantly higher parasitemia than Duffy-negative ones. The relatively low parasitemia suggests that although Duffy-negativity does not confer resistance to *P. vivax*^11,13,41^, it provides some level of protection against severe symptoms. This lower parasitemia in the Duffy-negative samples is likely what also caused the lower coverage in the whole genome sequencing samples as compared to the Duffy-positive ones^42^.

A significantly higher number of SNPs was detected in *P. vivax* infecting Duffy-positive compared to Duffy-negative individuals across all 14 chromosomes. For Duffy-positives, the highest SNP density was detected in chromosomes 3, 4, and 10 for synonymous SNPs and 2, 4, and 10 in nonsynonymous, which slightly contrasts with the Ethiopian and Southeast Asian isolates where chromosome 9 was most polymorphic, in addition to chromosomes 4 and 10^27^. For Duffy-negatives, the highest SNP density was detected in chromosomes 1, 2, and 4 for synonymous SNPs and 1, 5, and 10 for nonsynonymous ones. Only chromosome 4 for the synonymous SNPs and 10 for the nonsynonymous SNPs line up with the most polymorphic chromosomes in the Ethiopian and Southeast Asian isolates^27^. One potential reason for this discrepancy is that this study analyzed SNP density (i.e., SNPs per 1kb region) rather than total SNPs to account for differences in chromosome length. Some of the highly polymorphic chromosomes, such as chromosome 10, are due to polymorphisms in the *PvMSP* genes (*MSP*3G, *MSP*3.5, *MSP*3.8, *MSP*3.9, *MSP*3.10, and *MSP*3.11). *PvMSP3.8* is responsible for the highest number of nonsynonymous SNPs (38 SNPs) on chromosome 10, followed by *PvMSP3.9* and *PvMSP3.10*. The second highest number of nonsynonymous SNPs was observed in the *Plasmodium* interspersed repeat (*PIR*) gene. The P01 genome of *P. vivax* has over 1,200 *PIR* genes, and these genes are important in parasite survival, antigenic variation, and virulence. The *PIR* genes are known to be genetically diverse, which potentially indicates subfamilies with unique functions^43,44^. On chromosome 1, the *PIR* genes had the most nonsynonymous SNPs (43 and 32 SNPs) in Duffy-negatives. In chromosome 5, *RBP*2c had the most nonsynonymous SNPs in Duffy-negatives (65 SNPs). Since the *MSP* genes are expressed as immunogenic surface proteins, it is not surprising that these genes are highly polymorphic in the Sudanese *P. vivax* isolates^45,46^. High polymorphisms in *PIR* and *RBP*2c particularly in Duffy negative individuals may be indicative of selection for immune evasion, though low coverage across the Duffy-negative genomes prevented verification of this selection^47^. Further study should be done with more Duffy-negative samples with higher coverage to determine if there is selection pressure in these genes, potentially for alternative invasion. In the Duffy-positive samples, there is positive/directional selection pressure on 8 of the 44 EBP genes (*PvAMA1, PvDBP1, PvMSP3.5, PvMSP3.10, PvMSP5, PvRBP1a, PvRBP1b*, and *PvRBP2a*), indicating that the mutations in these genes are offering a fitness advantage to the parasite^48,49^. The 10 genes that have a dN/dS value of <1—*PvMAEBL, PvMSP1, PvMSP3.8, PvMSP3.9, PvMSP3G, PvMSP9, PvMSP10, PvTRAG3, PvTRAG14*, and *PvTRAG20*—may not necessarily be undergoing negative selection. This is because they are from the same population and dN/dS values less than 1 among a single population could indicate positive or negative selection^48,49^. More data from a separate population should be included in a future study to determine which of the two it is.

At the gene level, Duffy-positive *P. vivax* showed significantly higher nucleotide diversity across all 44 *PvEBP* genes compared to Duffy-negative ones. Low nucleotide diversity can be indicative of a selection sweep or smaller effective population size in the Duffy-negatives compared to the Duffy-positives^50,51^. In Sudan, like Ethiopia, about 30% of the general population is Duffy negative^33^. The smaller population size of Duffy-negative individuals may imply limited parasite reservoirs, resulting in lower polymorphisms^52^. Although our prior study showed that transmission could occur between Duffy-positive and Duffy-negative individuals and that Duffy-negatives in Sudan most often—but not always—serve as sink populations^32^, it is possible that only parasite strains carrying certain mutations in these erythrocyte binding genes can infect Duffy-negatives.

Compared to *PvDBP*1 and *PvRBP*2b, *PvEBP/DBP*2 was most conserved with only a single nonsynonymous SNP in one Duffy-negative isolate. *PvEBP/DBP*2 was previously shown to moderately bind to Duffy-negative erythrocytes^53,54^. Despite the conservation of amino acids in *PvEBP/DBP2*, we did not observe selection pressure on the gene, although this was influenced by many values of 0 for dS. Inclusion of more sequences with high coverage may help us obtain a more accurate, nonzero value. With all of the variation in *PvDBP1*, however, we did see slight positive selection, indicating that some of the amino acid mutations we found, such as Lys403Ser in the binding region, may confer a fitness advantage in transmission settings with high Duffy-negativity. Additionally, due to the large difference between lysine and serine residues, there is likely a large effect on the protein. Other amino acid changes in the binding region that may have a large effect present due to substantial differences in amino acid residues in both Duffy-positive and Duffy-negative samples include Lys327Glu, Trp393Arg, and Ile459Lys.

Furthermore, the three reticulocyte binding genes we analyzed on the amino acid level—*PvRBP1a, PvRBP1b*, and *PvRBP2a*—all showed evidence of positive selection with dN/dS values >1. Mutations in binding regions of *PvRBP1a* and *PvRBP2a* such as Asn362Thr and Glu439Gly, respectively, that are present in both Duffy-positive and Duffy-negative samples, may help to confer fitness advantages to spreading in a mixed Duffy-positive and Duffy-negative population, and may perhaps be implicated in alternative invasion in Duffy-negatives. In RBP2a, both Glu439Gly and Met512Lys—the two most prevalent amino acid mutations in Duffy-positive samples and in the binding region—are very large changes in residues that likely have a pronounced effect on binding. Though not present in Duffy-negative samples, mutations in RBP1a that are prevalent in the Duffy-positive group in the binding region with a large change in amino acid structure include Pro523Gln, Met552Thr, and Arg561Gly. These amino acid changes, which are all very different from the reference residue, may play a large part in the positive selection pressure on *PvRBP1a*, though further analysis is needed to verify this.

*PvMSP1*, with many mutations in Duffy-positive samples, was found to be under positive selection pressure. Although the mutations in this gene are not as prevalent as the other genes analyzed at the amino acid level, this one has the most mutations present. MSP1 is a protein with many repetitive elements, and polymorphic repetitive regions facilitate immune evasion, so the sheer number of amino acid changes present in this gene in Duffy-positives, alongside positive selection pressure, shows that this gene is under immune selective pressure^55^. Since there is only one amino acid change present in the Duffy-negative samples, it could indicate that either parasites with the mutations are unable to invade Duffy-negative reticulocytes or that there is not enough immune pressure on parasites in the Duffy-negative infecting parasite population to select for many mutants in this gene. More Duffy-negative samples with increased coverage are needed to verify the lack of mutants in *PvMSP1*.

*PvTRAg38*, potentially implicated in alternative invasion by binding to Band 3^20^, was found to have only two amino acid mutations. Even though these are not in the binding region, the Gln201Pro mutation, present in both Duffy-positive and Duffy-negative individuals, likely leads to a change in conformation due to proline’s unique structure. Furthermore, the Val271Glu mutation, also present in both Duffy-positive and Duffy-negative samples, represents a shift from a negative charge to a nonpolar residue. Both of these shifts could result in conformational changes, as, even if neither of the residues are in the binding region of TRAg38, they are still within the antigenic region^56^. Future directions to verify effects of all of the aforementioned conformational and functional changes include *in silico* protein structure analysis and *in vitro* binding assays.

## Conclusion

Among *P. vivax* infections in Sudan, a negative correlation was observed between parasitemia and age. A larger number of *P. vivax* infections were detected in males than females. Duffy-positive individuals had significantly higher parasitemia than Duffy-negative individuals. Genomic analyses of the 14 chromosomes indicated that *P. vivax* in Duffy-negatives was relatively conserved, compared to Duffy-positives that had much more genomic variation. The highly conserved *PvEBP/DBP*2 may imply selection pressure and potentially a key role in the invasion of Duffy negative erythrocytes. The prevalence of amino acid mutations of EBPs under positive selection pressure indicates beneficial effects to mutating residues in the binding or antigenic regions of these genes. Findings of this paper provide insights into epidemiological and genetic features of *P. vivax* in Duffy-positive and Duffy-negative Sudanese. These findings guide the way towards elucidating alternative invasion mechanisms of *P. vivax* in Duffy-negative individuals as well as highlighting mutations in genes that may be used as vaccine candidates.

## Methodology

### Ethics statement

Ethical clearance was obtained from Khartoum State Ministry of Health, Sudan, with the number KMOH-REC-062.2. Written informed consent was obtained from each participant and guardians of minors prior to participation in the study. All methods were performed in accordance with relevant guidelines and regulations.

### Sample collection and processing

#### Study sites and sample collection

A cross-sectional hospital-based study was conducted between May 2018 to December 2021 in hospitals and health facilities from Khartoum, Gezera Slang, Alsarorab, River Nile and El-Obeid. A total of 221 whole blood samples from suspected *P. vivax* patients in Khartoum (56), Gezira Slang (47), Alsarorab (33), River Nile (61), El-Obeid (22), and Halfa (2). *P. vivax* was diagnosed by microscopic examination of Giemsa-stained thin and thick blood films and/or rapid diagnosis test (SD Bioline, Standard Diagnostics Inc., South Korea). Demographical and clinical data were recorded using questionnaire including age, sex, tribes, and medical history. Patients who were infected with other *Plasmodium* species (*P. falciparum, P. malariae* and *P. ovale*), or otherwise unknown to be mono-infected, were excluded from this study’s epidemiological analysis alongside samples without a recorded parasitemia. After these filters, remaining were 190 samples for analysis from Khartoum (45), Gezera Slang (44), Alsarorab (33), River Nile (48), and El-Obeid (20; Figures S2A S3). Duffy-status was determined for 183 of the mono-infected samples (173 Duffy-positive, 10 Duffy-negative), age was recorded for 171 patients (157 Duffy-positive, 7 Duffy-negative, 7 unknown), and sex was recorded for 180 patients (164 Duffy-positive, 9 Duffy-negative, and 7 unknown; Figure S1). Missing Duffy-status data were manually excluded from relevant plots, while missing age and sex data were automatically excluded during software-based analysis.

#### Identification of malaria parasite species and parasite density using light microscopy

According to standard protocols, both thin and thick blood smears were stained with 3% freshly prepared Giemsa (RAL Diagnostics, France) and allowed to air dry at room temperature for one hour^57,58^. Thick blood films were used for detecting Plasmodium parasites, while thin blood films facilitated the identification of the infecting species. Parasite density was estimated by counting the number of parasites against 200 or 500 leukocytes, depending on the parasite load, with the assumption of a leukocyte density of 8,000 per μL^59^. Reported parasitemia was based on microscopy findings.

#### Identification of malaria parasite species using nested PCR

Genomic DNA was extracted from venous blood or dried blood spots (Whatman 3mm filter paper) using ZymoBead Genomic DNA kit (Zymo Research) following the manufacturer’s procedures^60^. The concentrations of DNA were determined using a Nanodrop spectrophotometer (UV1visible Nanodrop 1000, Thermo Fisher). The obtained DNA concentration ranged from 150–700 ng/μl. PCR was performed in total volume of 20μl using maxime PCR premix (intron biotechnology, Inc, South Korea) with i-Taq DNA polymerase. Species-specific primer pairs were used to amplify partial region of 18S rRNA gene which was used as a template for the second PCR round to detect *P. vivax* as described in previous study^61^. The first PCR condition was as follows: initial denaturation 94°C for 2 minutes, followed by 40 cycles of: denaturation 94°C for 30 seconds, annealing 55°C for 1 minute, extension 72°C for 1 minute and final extension 72°C for 5 minutes. The second PCR was as follows: initial denaturation 94°C for 2 minutes, followed by 40 cycles of: denaturation 94°C for 30 seconds, annealing 58°C for 1 minute, extension 72°C for 1 minute and a final extension 72°C for 5 minutes. Negative and positive controls were included in each PCR.

#### Quantification of *P. vivax* parasite using qPCR

SYBR Green qPCR method that amplifies a segment of the 18S rRNA genes of *P. vivax*^62^, was used to quantify parasite density of the samples. Amplification was performed in a 20μL reaction containing 10μL of 2x SYBR Green qPCR Master Mix (Thermo Scientific), 0.5 μM of primer (forward: 5’AGAATTTTCTCTTCGGAGTTTATTCTTAGATTGCT-3’; reverse: 5’GCCGCAAGCTCCACGCCTGGTGGTGC-3’), and 1μL of genomic DNA. PCR conditions were as follows: initial denaturation of 95°C for 3 minutes, followed by 45 cycles of: denaturation at 94°C for 30 seconds, annealing at 55°C for 30 seconds, extension at 68°C for 1 minute immediately followed by 95°C hold step for 10 seconds and a final melting curve step with temperatures increasing from 65°C to 95°C in 0.5°C increments. Each assay included a positive plasmid control for the 18S rRNA *P. vivax* gene (MRA-178; BEI Resources) to ensure primer specificity in addition to negative controls. A ten-fold dilution series was used to estimate the standard curve and thus amplification efficiency*. P. vivax* parasitemia was calculated with the following equation: Parasite DNA (per/μL) = [2^E×(40−Ctsample)^/10]; where Ct is the threshold cycle of the individual sample and E is the amplification efficiency^63^.

#### Duffy blood group genotyping using qPCR

All *P. vivax* positive samples were included in Duffy blood group genotyping based on TaqMan qPCR assays^64^. The primers (forward: 5’GGCCTGAGGCTTGTGCAGGCAG-3’; reverse: 5’ CATACTCACCCTGTGCAGACAG-3’) and dye-labeled probes (FAM: CCTTGGCTCTTA[C]CTTGGAAGCACAGG-BHQ; HEX: CCTTGGCTCTTA[T]CTTGGAAGCACAGG-BHQ) amplified the GATA1 transcription factor-binding site of the *DARC* gene promoter. Amplification was performed in a 20μL reaction containing 7μL TaqMan Fast Advanced Master mix (Thermo Scientific), 0.5 μM of forward and reverse primers, 0.5 μM of each of the dye-labeled probes, and 1μL of DNA template. PCR conditions were as follows: initial denaturation of 95°C for 2 minutes, followed by 45 cycles of: denaturation at 95°C for 3 seconds and annealing at 58°C for 30 seconds. The fluorescent signals emitted by the dye-labeled probes provided the data for allelic discrimination and determination of the Duffy genotype. The *DARC* gene from a subset of Duffy-positive (C/T and T/T) and of Duffy-negative (C/C) samples were amplified and sequenced to confirm the genotyping results. Samples that did not have Duffy-status associated with them were not included in Duffy comparison but were still included in other analysis.

#### Sequencing of the Duffy blood group genotyping

To determine the DARC status of the *P. vivax* infected individuals we used a protocol described by A. Chittoria et al.^65^. A 630 bp PCR fragment covering the promoter region was amplified to determine the presence of a C or T allele at the nucleotide position −33 downstream of the promoter region of the human Duffy gene^66^. The presence of only the C allele represents the lack of expression of the Duffy gene on the erythrocytes, which is also denoted as the FY*O homozygote genotype. The detection of both the C and T alleles represents the heterozygote condition, and the T allele indicates the absence of the FY*O genotype and normal expression of the Duffy gene. The primers used were: F-5’ TTTCCTGAGTGTAGTCCCAACC 3’ and R-5’ AAGGTCTCTGCAGGAGTCAGAT 3’. The PCR was performed using the Q5^®^ High-Fidelity DNA Polymerase (M0491S/L NEB). Amplification was performed in a 23μL reaction containing 5μl of 5X Q5 Reaction Buffer, 0,5μl of 10 mM dNTPs, 1.25μl of forward and reverse primers, 14.75μl of PCR grade water, 0.25 μl of Q5 High-Fidelity DNA Polymerase and a total of 2ul of DNA (between 1–10 ng/ul concentration) was used from each sample, and water was used as a negative control. PCR conditions were as follows: initial denaturation of 98°C for 30 seconds, followed by 25–35 cycles of: denaturation at 98°C for 10 seconds, annealing at 58°C for 30 seconds, extension at 72°C for 30 seconds and final extension at 72°C for 2 minutes. To confirm the correct size of the amplification, PCR products were run in a 1% agarose gel. The PCR products were then prepared for Sanger sequencing and were cleaned using a Qiagen QIAquick PCR Purification Kit. Dye-terminator sequencing was performed and products were analyzed on an ABI 377 or 3700 automated sequencer (performed at GeneWize). For nucleotide sequencing analysis, chromatograms were analyzed using the SnapeGene software. Sequences were compared to a previously published human Duffy gene reference obtained from NCBI (NG_011626.3 Homo sapiens atypical chemokine receptor 1 (Duffy blood group) (ACKR1), RefSeqGene (LRG_801) on chromosome 1).

#### Data collection and whole genome sequencing

A total of 30 whole blood samples were collected for whole genome sequencing. 15 of these samples were included in epidemiological analysis (12 Duffy-positive, 3 Duffy-negative), and 15 were excluded from it due to unknown coinfection status and parasitemia (8 Duffy-positive, 7 Duffy-negative). We used a Lymphoprep/Plasmodpur-based protocol for white blood cell depletion and red blood cell enrichment^67^. We then used Zymo Bead Genomic DNA kit (Zymo Research) to extract DNA from ~1 mL of red blood cell pellet. Of these, 15 of the samples underwent selective whole genome amplification (sWGA) before sequencing to increase yield.

Whole genome sequencing was performed at two different facilities with 15 samples sequenced on Illumina HiSeq3000 at the Wellcome Sanger Institute (European Nucleotide Archive [ENA], accession numbers in Supplemental Table 1). Whole genome sequencing of another 15 DNA samples was performed using the Illumina HiSeq platform at LSHTM. All sequencing data have been deposited in the ENA under project accession number PRJEB94030.

#### Quality control, raw reads processing and variant calling

Sequenced samples were aligned to the P01 reference genome downloaded from PlasmoDB release 68^43,68^ using BWA-mem version 0.7.18-r1243 with default parameters. The initial BAM files from the alignment were then processed using samtools v1.21, where reads were sorted by position and duplicate reads were removed using the fixmate and markdup functions.^69^. Single nucleotide polymorphisms (SNPs) and indels were then called using GATK v4.5.0.0’s HaplotypeCaller function to create individual variant files.^70^. The sample variant files were then either merged using CombineGVCFs or treated individually and variants were filtered using SelectVariants and VariantFiltration functions according to the following criteria: quality score > 30, quality normalized by read depth >2, Fisher Strand<60, mapping quality >40, mapping quality rank sum test >−12.5, ReadPos rank sum test >−8, and strand odds ratio <4. Only SNP data was included in the final variant file, with indels removed. The filtered population and individual variant files were annotated using snpEff v5.2e with gene annotation files, protein and CDS sequence data obtained from PlasmoDB^71^. The SNPs annotated as missense, start lost, nonsense, and nonstop were categorized as nonsynonymous mutations and all other annotations were categorized as synonymous mutations. Merged data were used in identifying top SNPs (Supplemental Table 5), all other SNPs were called from individual variant calls.

#### Amino acid prevalence and nucleotide diversity

Nucleotide diversity of 44 potential EBPs highlighted in Ford et al., 2020 was calculated using DnaSP version 6^72^. To remove introns and untranslated regions from the DNA sequences, we used the program AliView to view all samples aligned together with the reference sequence from PlasmoDB to identify the introns^73^. We assessed amino acid substitution rates of genes involved in host erythrocyte invasion where the binding region for these genes are known. We compared the amino acid sequences for *PvDBP*, *PvDBP2/EBP*, *PvRBP1a*, *PvRBP1b*, *PvRBP2a*, *PvRBP2a*, *PvMSP1* and *PvTRAg38* with the reference P01 sequence between Duffy negative and positive groups. Each observed substitution was recorded, and the corresponding relative frequency was calculated within each population. Any substitutions with more than 5% of samples containing the mutation in the Duffy positive group were saved, and these same positions were compared to mutations (if any) in the Duffy negative population.

#### Selection pressure analysis

To detect selection pressure in the aforementioned 44 erythrocyte binding genes, we calculated the ratio of nonsynonymous mutations to synonymous mutations (dN/dS) using the YN00 program from the PAML package, phylogenetic analysis by maximum likelihood, developed by Ziheng Yang^74–76^. The sites undergoing positive selection are defined as those with an elevated dN/dS ratio. This program was ran with the parameters icode=0, weighting=0, commonf3×4=0, and ndata=1. Pairwise dN and dS values across all samples using the Yang and Nielsen 2000 method^76^ were extracted and dN/dS was calculated for each pairwise sample grouping. From all pairwise samples, median dN/dS was recorded to omit the effects of outliers.

## Supplementary Material

Supplementary Files

This is a list of supplementary files associated with this preprint. Click to download.

- SupplementalTables.xlsx
- SupplementalFile1.docx

## Figures and Tables

**Table 1.** Median dN/dS per Gene

Gene	Duffy-positive	Duffy-negative
AMA1	1.406	0
DBP1	1.133	0
MAEBL	0.385	0
MSP1	0.757	0
MSP3.5	1.371	0
MSP3.8	0.767	0
MSP3.9	0.717	0
MSP3.10	1.140	0
MSP3G	0.568	0
MSP5	3.012	0
MSP9	0.756	0
MSP10	0.58	0
RBP1a	1.782	0
RBP1b	1.25	0
RBP2a	2.333	0
RON2	0.5	0
TRAG3	0.348	0
TRAG14	0.824	0

Median dN/dS per Erythrocyte Binding Gene candidate across Duffy-positive and Duffy-negative samples.

## Data Availability

Sequences of 15 genomes are available on the ENA accession browser according to accession numbers listed in Supplemental Table 1. Sequences of other 15 genomes have been deposited in the ENA under project accession number PRJEB94030.

## References

[1] Gartner V. Genomic insights into Plasmodium vivax population structure and diversity in central Africa. Malar J 23, 27 (2024). 10.1186/s12936-024-04852-y 38238806 PMC10797969

[2] Menegon M. Genetic diversity and population structure of Plasmodium vivax isolates from Sudan, Madagascar, French Guiana and Armenia. Infect Genet Evol 27, 244–249 (2014). 10.1016/j.meegid.2014.07.029 25102032

[3] Price R. N., Douglas N. M. & Anstey N. M. New developments in Plasmodium vivax malaria: severe disease and the rise of chloroquine resistance. Curr Opin Infect Dis 22, 430–435 (2009). 10.1097/QCO.0b013e32832f14c1 19571748

[4] Anstey N. M., Russell B., Yeo T. W. & Price R. N. The pathophysiology of vivax malaria. Trends Parasitol 25, 220–227 (2009). 10.1016/j.pt.2009.02.003 19349210

[5] Carvalho T. A. Plasmodium vivax infection in Anajas, State of Para: no differential resistance profile among Duffy-negative and Duffy-positive individuals. Malar J 11, 430 (2012). 10.1186/1475-2875-11-430 23259672 PMC3544589

[6] Howes R. E. The global distribution of the Duffy blood group. Nat Commun 2, 266 (2011). 10.1038/ncomms1265 21468018 PMC3074097

[7] Mendes C. Duffy negative antigen is no longer a barrier to Plasmodium vivax--molecular evidences from the African West Coast (Angola and Equatorial Guinea). PLoS Negl Trop Dis 5, e1192 (2011). 10.1371/journal.pntd.0001192 21713024 PMC3119644

[8] Barnwell J. W., Nichols M. E. & Rubinstein P. In vitro evaluation of the role of the Duffy blood group in erythrocyte invasion by Plasmodium vivax. J Exp Med 169, 1795–1802 (1989). 10.1084/jem.169.5.1795 2469769 PMC2189319

[9] Chitnis C. E. & Miller L. H. Identification of the erythrocyte binding domains of Plasmodium vivax and Plasmodium knowlesi proteins involved in erythrocyte invasion. J Exp Med 180, 497–506 (1994). 10.1084/jem.180.2.497 8046329 PMC2191600

[10] Ovchynnikova E. DARC extracellular domain remodeling in maturating reticulocytes explains Plasmodium vivax tropism. Blood 130, 1441–1444 (2017). 10.1182/blood-2017-03-774364 28754683 PMC5609335

[11] Miller L. H., Mason S. J., Clyde D. F. & McGinniss M. H. The resistance factor to Plasmodium vivax in blacks. The Duffy-blood-group genotype, FyFy. N Engl J Med 295, 302–304 (1976). 10.1056/NEJM197608052950602 778616

[12] Tournamille C., Colin Y., Cartron J. P. & Le Van Kim C. Disruption of a GATA motif in the Duffy gene promoter abolishes erythroid gene expression in Duffy-negative individuals. Nat Genet 10, 224–228 (1995). 10.1038/ng0695-224 7663520

[13] Menard D. Plasmodium vivax clinical malaria is commonly observed in Duffy-negative Malagasy people. Proc Natl Acad Sci U S A 107, 5967–5971 (2010). 10.1073/pnas.0912496107 20231434 PMC2851935

[14] Golassa L., Amenga-Etego L., Lo E. & Amambua-Ngwa A. The biology of unconventional invasion of Duffy-negative reticulocytes by Plasmodium vivax and its implication in malaria epidemiology and public health. Malar J 19, 299 (2020). 10.1186/s12936-020-03372-9 32831093 PMC7443611

[15] Popovici J., Roesch C. & Rougeron V. The enigmatic mechanisms by which Plasmodium vivax infects Duffy-negative individuals. PLoS Pathog 16, e1008258 (2020). 10.1371/journal.ppat.1008258 32078643 PMC7032691

[16] Verzier L. H., Coyle R., Singh S., Sanderson T. & Rayner J. C. Plasmodium knowlesi as a model system for characterising Plasmodium vivax drug resistance candidate genes. PLoS Negl Trop Dis 13, e0007470 (2019). 10.1371/journal.pntd.0007470 31158222 PMC6564043

[17] Ranjan A. & Chitnis C. E. Mapping regions containing binding residues within functional domains of Plasmodium vivax and Plasmodium knowlesi erythrocyte-binding proteins. Proc Natl Acad Sci U S A 96, 14067–14072 (1999). 10.1073/pnas.96.24.14067 10570199 PMC24191

[18] Mason S. J., Miller L. H., Shiroishi T., Dvorak J. A. & McGinniss M. H. The Duffy blood group determinants: their role in the susceptibility of human and animal erythrocytes to Plasmodium knowlesi malaria. Br J Haematol 36, 327–335 (1977). 10.1111/j.1365-2141.1977.tb00656.x 70210

[19] Bouyssou I. Unveiling P. vivax invasion pathways in Duffy-negative individuals. Cell Host Microbe 31, 2080–2092 e2085 (2023). 10.1016/j.chom.2023.11.007 38056460 PMC10727064

[20] De Meulenaere K. Band 3-mediated Plasmodium vivax invasion is associated with transcriptional variation in PvTRAg genes. Front Cell Infect Microbiol 12, 1011692 (2022). 10.3389/fcimb.2022.1011692 36250048 PMC9563252

[21] Gruszczyk J. Transferrin receptor 1 is a reticulocyte-specific receptor for Plasmodium vivax. Science 359, 48–55 (2018). 10.1126/science.aan1078 29302006 PMC5788258

[22] Kanjee U. Plasmodium vivax Strains Use Alternative Pathways for Invasion. J Infect Dis 223, 1817–1821 (2021). 10.1093/infdis/jiaa592 32941614 PMC8161644

[23] Malleret B. Plasmodium vivax binds host CD98hc (SLC3A2) to enter immature red blood cells. Nat Microbiol 6, 991–999 (2021). 10.1038/s41564-021-00939-3 34294905

[24] Lee S. K. Complement receptor 1 is the human erythrocyte receptor for Plasmodium vivax erythrocyte binding protein. Proc Natl Acad Sci U S A 121, e2316304121 (2024). 10.1073/pnas.2316304121 38261617 PMC10835065

[25] Drew D. R. Defining species-specific and conserved interactions of apical membrane protein 1 during erythrocyte invasion in malaria to inform multi-species vaccines. Cell Mol Life Sci 80, 74 (2023). 10.1007/s00018-023-04712-z 36847896 PMC9969379

[26] Yap A. Conditional expression of apical membrane antigen 1 in Plasmodium falciparum shows it is required for erythrocyte invasion by merozoites. Cell Microbiol 16, 642–656 (2014). 10.1111/cmi.12287 24571085 PMC4231980

[27] Ford A. Whole genome sequencing of Plasmodium vivax isolates reveals frequent sequence and structural polymorphisms in erythrocyte binding genes. PLoS Negl Trop Dis 14, e0008234 (2020). 10.1371/journal.pntd.0008234 33044985 PMC7581005

[28] Ngwana-Joseph G. C. Genomic analysis of global Plasmodium vivax populations reveals insights into the evolution of drug resistance. Nat Commun 15, 10771 (2024). 10.1038/s41467-024-54964-x 39738010 PMC11685768

[29] Jennison C. Plasmodium vivax populations are more genetically diverse and less structured than sympatric Plasmodium falciparum populations. PLoS Negl Trop Dis 9, e0003634 (2015). 10.1371/journal.pntd.0003634 25874894 PMC4398418

[30] Neafsey D. E. The malaria parasite Plasmodium vivax exhibits greater genetic diversity than Plasmodium falciparum. Nat Genet 44, 1046–1050 (2012). 10.1038/ng.2373 22863733 PMC3432710

[31] Benavente E. D. Distinctive genetic structure and selection patterns in Plasmodium vivax from South Asia and East Africa. Nat Commun 12, 3160 (2021). 10.1038/s41467-021-23422-3 34039976 PMC8154914

[32] Kepple D. Plasmodium vivax From Duffy-Negative and Duffy-Positive Individuals Share Similar Gene Pools in East Africa. J Infect Dis 224, 1422–1431 (2021). 10.1093/infdis/jiab063 33534886 PMC8557672

[33] Lo E. Contrasting epidemiology and genetic variation of Plasmodium vivax infecting Duffy-negative individuals across Africa. Int J Infect Dis 108, 63–71 (2021). 10.1016/j.ijid.2021.05.009 33991680 PMC9004484

[34] Ryan J. R. Evidence for transmission of Plasmodium vivax among a duffy antigen negative population in Western Kenya. Am J Trop Med Hyg 75, 575–581 (2006). 17038676

[35] World Health Organization. World malaria report 2024: addressing inequity in the global malaria response. (Geneva, 2024).

[36] Howes R. E. Global Epidemiology of Plasmodium vivax. Am J Trop Med Hyg 95, 15–34 (2016). 10.4269/ajtmh.16-0141 PMC5198891 27402513

[37] Schumacher R. F. & Spinelli E. Malaria in children. Mediterr J Hematol Infect Dis 4, e2012073 (2012). 10.4084/MJHID.2012.073 23205261 PMC3507524

[38] Koepfli C. Blood-Stage Parasitaemia and Age Determine Plasmodium falciparum and P. vivax Gametocytaemia in Papua New Guinea. PLoS One 10, e0126747 (2015). 10.1371/journal.pone.0126747 25996916 PMC4440770

[39] Lin E. Differential patterns of infection and disease with P. falciparum and P. vivax in young Papua New Guinean children. PLoS One 5, e9047 (2010). 10.1371/journal.pone.0009047 20140220 PMC2816213

[40] Tjitra E. Multidrug-resistant Plasmodium vivax associated with severe and fatal malaria: a prospective study in Papua, Indonesia. PLoS Med 5, e128 (2008). 10.1371/journal.pmed.0050128 18563962 PMC2429950

[41] Albsheer M. M. A. Distribution of Duffy Phenotypes among Plasmodium vivax Infections in Sudan. Genes (Basel) 10 (2019). 10.3390/genes10060437 PMC6628573 31181786

[42] Ibrahim A. Selective whole genome amplification of Plasmodium malariae DNA from clinical samples reveals insights into population structure. Sci Rep 10, 10832 (2020). 10.1038/s41598-020-67568-4 32616738 PMC7331648

[43] Auburn S. A new Plasmodium vivax reference sequence with improved assembly of the subtelomeres reveals an abundance of pir genes. Wellcome Open Res 1, 4 (2016). 10.12688/wellcomeopenres.9876.1 28008421 PMC5172418

[44] Carlton J. M. Comparative genomics of the neglected human malaria parasite Plasmodium vivax. Nature 455, 757–763 (2008). 10.1038/nature07327 18843361 PMC2651158

[45] Sachdeva S., Ahmad G., Malhotra P., Mukherjee P. & Chauhan V. S. Comparison of immunogenicities of recombinant Plasmodium vivax merozoite surface protein 1 19- and 42-kiloDalton fragments expressed in Escherichia coli. Infect Immun 72, 5775–5782 (2004). 10.1128/IAI.72.10.5775-5782.2004 15385477 PMC517592

[46] Fernandez-Becerra C. Naturally-acquired humoral immune responses against the N- and C-termini of the Plasmodium vivax MSP1 protein in endemic regions of Brazil and Papua New Guinea using a multiplex assay. Malar J 9, 29 (2010). 10.1186/1475-2875-9-29 20092651 PMC2835717

[47] Gupta E. D. Naturally Acquired Human Antibodies Against Reticulocyte-Binding Domains of Plasmodium vivax Proteins, PvRBP2c and PvRBP1a, Exhibit Binding-Inhibitory Activity. J Infect Dis 215, 1558–1568 (2017). 10.1093/infdis/jix170 28379500 PMC5853946

[48] Goodswen S. J., Kennedy P. J. & Ellis J. T. A Gene-Based Positive Selection Detection Approach to Identify Vaccine Candidates Using Toxoplasma gondii as a Test Case Protozoan Pathogen. Frontiers in Genetics Volume 9 – 2018 (2018). 10.3389/fgene.2018.00332 PMC6109633 30177953

[49] Kryazhimskiy S. & Plotkin J. B. The population genetics of dN/dS. PLoS Genet 4, e1000304 (2008). 10.1371/journal.pgen.1000304 19081788 PMC2596312

[50] VanBuren R. Extremely low nucleotide diversity in the X-linked region of papaya caused by a strong selective sweep. Genome Biol 17, 230 (2016). 10.1186/s13059-016-1095-9 27890017 PMC5125041

[51] Arnott A. Global Population Structure of the Genes Encoding the Malaria Vaccine Candidate, Plasmodium vivax Apical Membrane Antigen 1 (PvAMA1). PLoS Negl Trop Dis 7, e2506 (2013). 10.1371/journal.pntd.0002506 24205419 PMC3814406

[52] Mwesigwa A. Genetic diversity and population structure of Plasmodium falciparum across areas of varied malaria transmission intensities in Uganda. Malar J 24, 97 (2025). 10.1186/s12936-025-05325-6 40128854 PMC11934718

[53] Hester J. De novo assembly of a field isolate genome reveals novel Plasmodium vivax erythrocyte invasion genes. PLoS Negl Trop Dis 7, e2569 (2013). 10.1371/journal.pntd.0002569 24340114 PMC3854868

[54] Ntumngia F. B. A Novel Erythrocyte Binding Protein of Plasmodium vivax Suggests an Alternate Invasion Pathway into Duffy-Positive Reticulocytes. mBio 7 (2016). 10.1128/mBio.01261-16 PMC4999553 27555313

[55] Ferreira M. U., da Silva Nunes M. & Wunderlich G. Antigenic diversity and immune evasion by malaria parasites. Clin Diagn Lab Immunol 11, 987–995 (2004). 10.1128/cdli.11.6.987-995.2004 15539495 PMC524792

[56] Consortium T. U. UniProt: the Universal Protein Knowledgebase in 2025. Nucleic Acids Research 53, D609–D617 (2024). 10.1093/nar/gkae1010 PMC11701636 39552041

[57] World Health Organization. Malaria microscopy quality assurance manual-version 2. (2016).

[58] Makler M. T., Palmer C. J. & Ager A. L. A review of practical techniques for the diagnosis of malaria. Ann Trop Med Parasitol 92, 419–433 (1998). 10.1080/00034989859401 9683894

[59] Hamid M. M. A. Diagnostic accuracy of an automated microscope solution (miLab) in detecting malaria parasites in symptomatic patients at point-of-care in Sudan: a case-control study. Malar J 23, 200 (2024). 10.1186/s12936-024-05029-3 38943203 PMC11212432

[60] Lo E. Frequent expansion of Plasmodium vivax Duffy Binding Protein in Ethiopia and its epidemiological significance. PLoS Negl Trop Dis 13, e0007222 (2019). 10.1371/journal.pntd.0007222 31509523 PMC6756552

[61] Snounou G. Detection and identification of the four malaria parasite species infecting humans by PCR amplification. Methods Mol Biol 50, 263–291 (1996). 10.1385/0-89603-323-6:263 8751365

[62] Lo E. Molecular epidemiology of Plasmodium vivax and Plasmodium falciparum malaria among Duffy-positive and Duffy-negative populations in Ethiopia. Malar J 14, 84 (2015). 10.1186/s12936-015-0596-4 25884875 PMC4340780

[63] Murphy S. C. Real-time quantitative reverse transcription PCR for monitoring of blood-stage Plasmodium falciparum infections in malaria human challenge trials. Am J Trop Med Hyg 86, 383–394 (2012). 10.4269/ajtmh.2012.10-0658 22403305 PMC3284350

[64] Ahmed S. Prevalence and distribution of Plasmodium vivax Duffy Binding Protein gene duplications in Sudan. PLoS One 18, e0287668 (2023). 10.1371/journal.pone.0287668 37471337 PMC10358875

[65] Chittoria A., Mohanty S., Jaiswal Y. K. & Das A. Natural selection mediated association of the Duffy (FY) gene polymorphisms with Plasmodium vivax malaria in India. PLoS One 7, e45219 (2012). 10.1371/journal.pone.0045219 23028857 PMC3448599

[66] Pogo A. O. & Chaudhuri A. The Duffy protein: a malarial and chemokine receptor. Semin Hematol 37, 122–129 (2000). 10.1016/s0037-1963(00)90037-4 10791881

[67] Auburn S. An effective method to purify Plasmodium falciparum DNA directly from clinical blood samples for whole genome high-throughput sequencing. PLoS One 6, e22213 (2011). 10.1371/journal.pone.0022213 21789235 PMC3138765

[68] PlasmoDB: An integrative database of the Plasmodium falciparum genome. Tools for accessing and analyzing finished and unfinished sequence data. The Plasmodium Genome Database Collaborative. Nucleic Acids Res 29, 66–69 (2001). 10.1093/nar/29.1.66 11125051 PMC29846

[69] Danecek P. Twelve years of SAMtools and BCFtools. Gigascience 10 (2021). 10.1093/gigascience/giab008 PMC7931819 33590861

[70] Van der Auwera G. & O’Connor B. Genomics in the Cloud: Using Docker, GATK, and WDL in Terra (1st Edition). (O’Reilly Media, Inc., 2020).

[71] Cingolani P. A program for annotating and predicting the effects of single nucleotide polymorphisms, SnpEff: SNPs in the genome of Drosophila melanogaster strain w1118; iso-2; iso-3. Fly (Austin) 6, 80–92 (2012). 10.4161/fly.19695 22728672 PMC3679285

[72] Rozas J. DnaSP 6: DNA Sequence Polymorphism Analysis of Large Data Sets. Molecular Biology and Evolution 34, 3299–3302 (2017). 10.1093/molbev/msx248 29029172

[73] Larsson A. AliView: a fast and lightweight alignment viewer and editor for large datasets. Bioinformatics 30, 3276–3278 (2014). 10.1093/bioinformatics/btu531 25095880 PMC4221126

[74] Yang Z. PAML: a program package for phylogenetic analysis by maximum likelihood. Comput. Appl. Biosci. 13, 555–556 (1997). 9367129 10.1093/bioinformatics/13.5.555

[75] Yang Z. PAML 4: Phylogenetic Analysis by Maximum Likelihood. Mol. Biol. Evol. 24, 1586–1591 (2007). 17483113 10.1093/molbev/msm088

[76] Yang Z. & Nielsen R. Estimating synonymous and nonsynonymous substitution rates under realistic evolutionary models. Mol. Biol. Evol. 17, 32–43 (2000). 10666704 10.1093/oxfordjournals.molbev.a026236

